# Retroviral gene therapy in Germany with a view on previous experience and future perspectives

**DOI:** 10.1038/s41434-021-00237-x

**Published:** 2021-03-22

**Authors:** Michael A. Morgan, Melanie Galla, Manuel Grez, Boris Fehse, Axel Schambach

**Affiliations:** 1 Institute of Experimental Hematology, Hannover Medical School, Hannover, Germany; 2 REBIRTH Research Center for Translational Regenerative Medicine, Hannover Medical School, Hannover, Germany; 3 Institute for Tumor Biology and Experimental Therapy, Georg-Speyer-Haus, Frankfurt, Germany; 4 Research Department Cell and Gene Therapy, Department of Stem Cell Transplantation, University Medical Center Hamburg-Eppendorf, Hamburg, Germany; 5 Division of Hematology/Oncology, Boston Children’s Hospital, Harvard Medical School, Boston, MA USA

**Keywords:** Gene expression, Haematological diseases

## Abstract

Gene therapy can be used to restore cell function in monogenic disorders or to endow cells with new capabilities, such as improved killing of cancer cells, expression of suicide genes for controlled elimination of cell populations, or protection against chemotherapy or viral infection. While gene therapies were originally most often used to treat monogenic diseases and to improve hematopoietic stem cell transplantation outcome, the advent of genetically modified immune cell therapies, such as chimeric antigen receptor modified T cells, has contributed to the increased numbers of patients treated with gene and cell therapies. The advancement of gene therapy with integrating retroviral vectors continues to depend upon world-wide efforts. As the topic of this special issue is “Spotlight on Germany,” the goal of this review is to provide an overview of contributions to this field made by German clinical and research institutions. Research groups in Germany made, and continue to make, important contributions to the development of gene therapy, including design of vectors and transduction protocols for improved cell modification, methods to assess gene therapy vector efficacy and safety (e.g., clonal imbalance, insertion sites), as well as in the design and conduction of clinical gene therapy trials.

## Introduction

Gene therapy is a molecular medicine approach that can be used to treat patients with inherited diseases, such as those caused by gene defects in monogenic diseases, as well as to treat acquired diseases, such as cancer and severe infections. This may involve addition/replacement of missing genes, transfer of corrected or protective genes, repair of defective genes, or removal of disease-causing genes.

German physicians and scientists have a long history of contribution to gene therapy that can be traced back to the fundamental findings of Walther Flemming [1], whose microscopic analyses of cell division in the 1870s led him to coin the term “mitosis,” in which he described chromatin changes during nuclear division. This seminal discovery laid the foundation for later work by Avery, McLeod, and McCarty that revolutionized modern biology by demonstrating that DNA, and not proteins as widely believed at the time, is responsible for transfer of genetic traits [2–4].

The concept of treating inherited diseases by gene therapy was developed in the mid-1960s by Nobel laureates J. Lederberg and E. Tatum [5]. Soon thereafter and even before the first human gene was cloned, first attempts to use gene therapy in the clinics were made. In fact, already in 1970 a relatively unknown (since unsuccessful) attempt to treat inherited arginase deficiency using Shope papilloma virus was carried out in Cologne [6]. The therapeutic concept was based on the idea that the disease might be ameliorated by the viral arginase. This followed observations of long-term decreased serum arginine by E. Shope and colleagues after infection with the virus. However, no such effect was found in the two girls treated in Cologne.

Advances in modern genetics have greatly simplified the detection and characterization of patient genomes, thus allowing more precise diagnosis of genetic diseases. For example, global efforts such as the Human Genome Project coupled with next-generation sequencing technologies allow routine use of whole-genome and exome sequencing to interrogate patient genomes. Currently, there are an array of monogenic diseases (https://www.omim.org/) known to occur due to gene mutations that lead to loss of proteins or production of proteins with altered function, such as severe combined immunodeficiencies (SCIDs), Wiskott–Aldrich syndrome (WAS), chronic granulomatous disease (CGD), cerebral adrenoleukodystrophy (CALD), metachromatic leukodystrophy, hemoglobinopathies, like β-thalassemia and sickle cell disease (SCD), and epidermolysis bullosa among others. The elucidation of mechanisms that retroviruses use to infect cells, coupled with the advent of molecular biology, were important milestones that helped to develop gene transfer technologies that made the field of gene therapy possible. As the goal of gene therapy is transfer and expression of the therapeutic gene and not of the viral genes, processes such as vectorization of murine Moloney leukemia virus (MoMLV) and later human immunodeficiency virus type 1 (HIV-1) were critical to generate gene therapy vectors for gammaretro- and lentiviral-based gene therapeutic approaches. In the following sections, we highlight the contribution of German research groups to the vectorization of retroviruses, advances in cell modification protocols, improved control of transgene expression, analyses of vector integration sites and their impact on retroviral safety as well as translation of these technologies to clinical application.

### Retroviral vector evolution over time

Retroviral vectors have commonly been used to modify cells for gene therapy. They were derived from natural retroviruses that have evolved gene transfer mechanisms over millions of years. For example, vector systems based upon MoMLV (gammaretroviral vectors) and HIV (lentiviral vectors) are often used due to their capacities for efficient gene transfer and their property to integrate into the cell genome, thus allowing a stable genetic modification of the target cell. Successful gene therapy with retroviral vectors requires robust production of appropriately high levels of infectious retroviral particles (referred to as retroviral vector titer), efficient entry of retroviral particles into target cells (proper envelope glycoproteins used to pseudotype retroviral vector particles), and achievement of transgene expression levels that are high enough to elicit a therapeutic effect without undesired toxicity (e.g., due to transgene or retroviral vector insertion into the host cell genome).

Seminal work on the characterization and vectorization of Moloney viruses came from the Heinrich Pette Institute in Hamburg and is associated with work from Rudi Jaenisch, Christopher Baum, Manuel Grez, and Wolfram Ostertag [7, 8]. Initial γ-retroviral vector systems exploited the viral promoter and enhancer elements in the long terminal repeats (LTR) to express the therapeutic gene. One of the most popular vector constructs for gene therapy in the 1990s, the Murine stem cell virus [9], was based on the murine embryonic stem cell virus (MESV) first cloned in the Ostertag lab [10]. Subsequently, many efforts were directed toward optimizing retroviral vector systems to achieve high transgene expression levels thought to be necessary to achieve therapeutic efficacy. In one approach, modular vector systems were generated to ease exploration of genetic elements that influence transgene transcription and expression in target cells as well as development of gammaretroviral vector configurations with improved transgene expression [11–13]. For example, direct comparison of transgene expression levels produced from MoMLV and Moloney murine sarcoma virus vectors with those generated from FMEV vectors, which combine enhancer and promoter elements from the LTR from Friend mink cell focus-forming viruses (FMCF) with the 5′ untranslated leader region of MESV, demonstrated greatly enhanced (up to two orders of magnitude) transgene expression mediated by the FMEV vectors [14]. Modification of the standard MoMLV vector by incorporation of the LTR of the myeloproliferative sarcoma virus (MPSV) or 3′ LTR of the spleen focus-forming virus (SFFVp) in combination with a modified 5′-untranslated region (e.g., derived from the leader of the MESV) led to generation of gammaretroviral vectors devoid of viral coding sequences (with a so-called gag-frame leader) and with improved transgene expression levels (e.g., vectors with LTRs from MPSV: MP110, MP11, MP71, and MP91 or from SFFV: SF110, SF11, SF71, and SF91) [15].

**Fig. 1:**
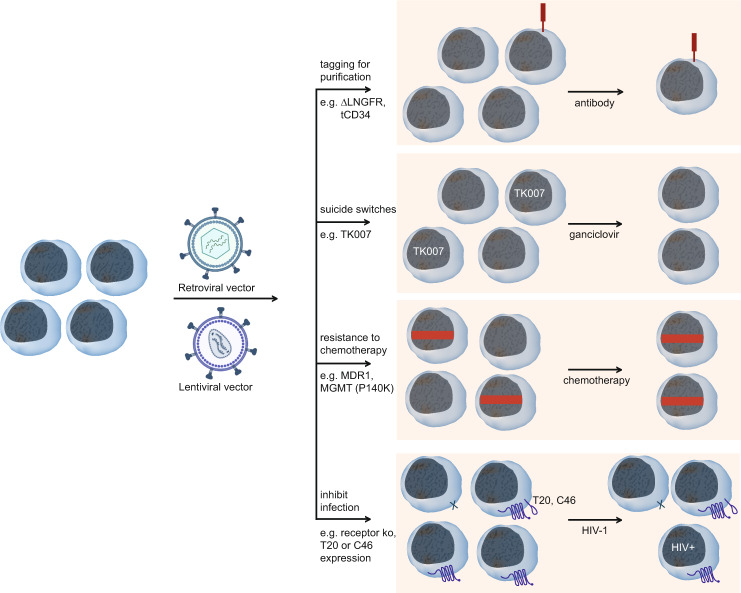
Several mechanisms can be employed to enrich gammaretroviral and lentiviral vector-modified cells in vitro or in vivo. For example, antibodies can be used to purify modified cells transduced with vectors designed to express truncated forms of CD34 (tCD34) or the low-affinity nerve growth receptor (ΔLNGFR). Cells can be engineered to express suicide genes such as the Herpes simplex virus thymidine kinase (scHSVtk) or variants (e.g., TK007) to allow elimination of cells in the case of adverse events. Cells can also be modified to express the multidrug resistance protein MDR-1 or the methylguanine methyltransferase P140K mutant (MGMT^P140K^) to endow improved resistance against medications such as chemotherapy so that only the modified cells (cells with the red rectangle) persist upon drug treatment. Knockout of receptors like CCR5 and CXCR4 can protect cells from HIV-1 infection. Furthermore, modification of cells to express small membrane-bound C peptides such as T20 and C46 can also prevent HIV-1 infection of modified cells. Retroviral particles were created with Biorender.com.

Efforts to generate vector systems that allow high transgene expression levels in early myeloid progenitors showed that different retroviral U3 regions led to preferred expression in lymphoid or myeloid hematopoietic cells and resulted in development of FMEV (FMCF/MESV hybrid vector) and MPEV (MPSV/MESV hybrid vector) [12]. Proof-of-concept experiments showed that these retroviral vectors could be used to express the multidrug resistance protein 1 (*mdr-1*) in hematopoietic progenitor cells to allow broader use of chemotherapy in cancer patients without the side effect of toxicity to the hematopoietic system [11, 12] (Fig. 1). Comparison of the effects of *cis*-acting modules such as splice sites, retroviral constitutive RNA transport elements, and the woodchuck hepatitis virus posttranscriptional regulatory element to improve transgene expression revealed that choice of the optimal module or combination of modules is highly dependent upon the transgene to be expressed [16].

While this strategy has the potential advantage of achieving strong expression levels of the transgene cargo, unfortunately, cell transformation was observed in some studies that used LTR-driven vectors to deliver therapeutic genes to hematopoietic stem cells (HSC) [17–20], whereas no such adverse events were observed upon transfer of transduced T cells to animal models [21, 22] and clinical trials that used similar vector configurations to modify terminally differentiated somatic cells such as T cells [21, 22]. Subsequent analyses showed that the transformation caused by the LTR-driven gammaretroviral gene therapy vector used to modify HSC was at least partially due to enhanced proto-oncogene (e.g., *LMO2*, *CCND2*, *MDS1*/*EVI1*, *PRDM16*) expression levels via the combination of the retroviral vector insertion site loci and the strong viral vector promoters and enhancers [23–25]. This process of transformation by transactivation of native genes caused by the retroviral vector is termed insertional mutagenesis. A retroviral vector system designed to minimize the risk of adverse events such as insertional mutagenesis and to allow the opportunity for cell-type-specific gene expression by deletion of the strong viral promoter and enhancer elements from the 3′-LTR (in so-called self-inactivating (SIN) vectors) was described earlier [26]. In these SIN vectors, therapeutic gene expression is accomplished via internal promoters that, in principle, could result in more physiological and optionally also cell-type-specific gene expression levels without transactivation of native genes in the modified cells [26].

Although gammaretroviral SIN vectors reduced the risk of insertional mutagenesis, one disadvantage was the low viral vector titers produced from the original SIN vector configurations. Exchange of the MPSV promoter for the Rous sarcoma virus (RSV) promoter to control expression of the full-length viral vector RNA in the packaging cells greatly improved viral vector titer production, which was even further enhanced by insertion of the SV40 enhancer upstream of the RSV promoter [27]. The principles used to generate gammaretroviral SIN vectors [26] were also successfully transferred to lentiviral vectors [28] and incorporation of SIN vectors into more recent clinical gene therapy trials seems to have mitigated the occurrence of insertional mutagenesis [29–35].

Naldini, Trono, and colleagues contributed pivotal work that led to development of the basic construction of lentiviral vectors currently used in clinical trials. In vivo application of first-generation lentiviral vectors that were based on HIV-1 and contained all HIV-1 proteins in the packaging unit, with the exception that the envelope protein was excluded, led to sustained expression of the transgene beta-galactosidase in neurons in the brains of adult rats [36]. Subsequent work showed that transgene transfer was still possible after deletion of the virulence genes *vpr*, *vif*, *vpu*, and *nef* from lentiviral vectors, which further improved the safety of lentiviral vector technology [37]. Modification of the 5′ LTR to contain a constitutively active RSV enhancer/promoter to drive transcription allowed elimination of the transactivator *Tat*, resulting in the third generation of lentiviral vectors and respective packaging system, which contain only *gag*, *pol*, and *rev* from the HIV-1 genome [38].

In contrast to gammaretroviral vectors, lentiviral vectors can transduce non-cycling cells [39], which is crucial for some highly relevant target cell populations such as hepatocytes and neurons. Analogously with gammaretroviral vectors, incorporation of the SIN principles further improved the safety of lentiviral vectors. These lentiviral SIN vector systems are currently the most widely applied integrating vector system, and are used in several clinical applications in Germany as further detailed below in the clinical trial section.

### Glycoprotein engineering to improve transfer and targeting of retroviral vectors

Retroviral vectors gain access to cells via interaction of glycoproteins expressed on the viral envelope with specific receptors present on the surface of the cell targeted for infection. Thus, in addition to the optimization of the retroviral vector genome architecture, the choice of glycoprotein used to pseudotype retroviral vectors can impact the efficacy of gene transfer to the target cell population. Several glycoproteins have been engineered from native viruses, with glycoproteins derived from gibbon ape leukemia virus (GALV) and vesicular stomatitis virus G (VSV-G) most commonly used in retroviral gene therapy protocols. Indeed, use of the VSV-G protein to pseudotype retroviral vectors increased the stability and broadened the host range of retroviral vectors [40]. At the same time, vectors pseudotyped with the GALV envelope were found to be particularly efficient for primary human T cells [41, 42].

The development of more effective and the possibility for engineering targeted envelope proteins remain important aspects for continued improvement of retroviral vector gene therapy approaches. In this direction, von Laer’s group showed that gammaretroviral and lentiviral vectors pseudotyped with the lymphocytic choriomeningitis virus (LCMV) glycoprotein could be efficiently generated, were highly stable, and exhibited a broad host range that allowed transduction of cell lines derived from different species, including human tissues, with less toxicity as compared to VSV-G pseudotyped retroviral vectors [43, 44]. The Buchholz group (Langen) has shown efficient and stable gene transfer mediated by lentiviral vectors pseudotyped with engineered glycoproteins from measles virus (MV) and Nipah virus, which can be exploited for cell-specific targeting. For example, that group generated lentiviral vectors pseudotyped with a modified MV glycoprotein that specifically targeted cells expressing the epidermal growth factor receptor (EGFR) or the B-lymphocyte antigen CD20. This was achieved via the incorporation of either EGF or an anti-CD20 single-chain antibody fragment into a truncated cytoplasmic tail of an H protein variant [45–47]. The same concept was applied for several other targets of clinical interest, e.g. CD4 and CD8 [48], glutamate receptor 4, epithelial cell adhesion molecule, CD105, NKp46 [49], CD30 [50], CD133 [51], CD19 [52], and murine major histocompatibility complex class II [53].

Another aspect critical to the long-term success of gammaretroviral and lentiviral vector-mediated gene therapy is stable integration into the host genome. Interestingly, gammaretroviral and lentiviral vectors differ in regard to preferred integration sites, with integration of gammaretroviral vectors more frequently in transcription start sites and lentiviral vectors in active genes. The exciting discoveries that distinct cellular factors seem to function in concert with viral integrases to direct insertion of gammaretroviral or lentiviral vector DNA into the host genome may be exploited to develop even safer gene therapy vectors [54–59]. The transcription factor lens epithelium-derived growth factor (p75) was shown to form complexes with HIV-1 intasomes (complexes between integrase and viral DNA) and direct them to active gene sites. For gammaretroviral MLV vectors, bromodomain and extraterminal domain (BET) proteins (especially Brd4) were shown to be tethering factors that influence the integrome [55–57]. Inhibition of BET protein expression, addition of BET inhibitors, or BET protein interaction with modified histone sites reduced MLV integration into transcription start sites [55–57].

Continued advances in these directions will allow improved vector design for targeting away from common integration sites and thus amelioration of potential toxic side effects of retroviral gene therapy [55, 58, 59].

### Characterization of retroviral vector insertion sites and improved biosafety assays

As with any therapeutic drug, detailed analyses are necessary to understand intended as well as unintended effects in order to achieve better insight into therapeutic mechanisms that may be exploited to improve treatment modalities. In the case of cells modified with retroviral gene therapy vectors, critical points with direct influence on therapeutic success beyond the gene transfer efficiency discussed above include the persistence and function of the modified cell therapy. Since retroviral vectors, like their natural counterparts, insert their genetic cargo into the genome of infected cells, development of techniques to characterize retroviral vector insertion sites was, and continues to be, an important enterprise in the gene therapy field. The von Kalle/Schmidt lab developed the widely applicable extension primer tag selection followed by solid-phase ligation-mediated polymerase chain reaction technique to characterize multiple rare gammaretroviral as well as lentiviral insertion sites with direct genomic sequencing [60]. Coupled with developments such as next-generation sequencing protocols [61, 62], improved ability to assess retroviral insertion sites represent important advances that helped to better understand potential side effects of gene therapy and are now used in routine monitoring of clinical trials [60, 63, 64].

The in vitro immortalization (IVIM) assay developed in the Baum laboratory (by Baum, Modlich, and coworkers) is a powerful preclinical method that is accepted by regulatory authorities to evaluate the safety of retroviral vector configurations [65–67]. The IVIM assay was developed following observations by Du et al. [68] and is based on transformation of murine bone marrow cells, which is quantified by generation of strongly replicating clones. This tool can be used to assess effects of vector elements, including viral promoters and enhancers, internal promoters, transgenes, and posttranscriptional regulatory elements. The IVIM assay was used to assess the transforming potential of several vectors developed to treat X-SCID, Artemis-SCID, recombinase-activating gene-1 SCID, X-CGD, HIV, SCD, severe hemoglobinopathies such as β-thalassemia, Fanconi anemia, infantile malignant osteopetrosis, acute myeloid leukemia (AML), adrenoleukodystrophy, Gaucher disease, Fabry disease, cystinosis, and familial hemophagocytic lymphohistiocytosis [69–78] (and personal communication from Dr. Michael Rothe (MHH)).

In addition to gammaretroviral and lentiviral vector systems, an alpharetroviral vector system was more recently developed for gene therapy applications [79, 80]. Evaluation of integration sites showed that the alpharetroviral vector has a more neutral integration pattern than gammaretroviral and lentiviral vectors [79–81]. IVIM assays also demonstrated differences among SIN retroviral vector systems engineered with internal promoters to drive transgene expression, with fewer transformation events in murine bone marrow cells transduced with alpharetroviral SIN vectors as compared to gammaretroviral and lentiviral SIN vectors [79].

## Gene therapy for monogenic diseases

### Severe combined immunodeficiency (SCID)

SCID patients have gene defects that result in loss of immunity (e.g., due to T, natural killer (NK), and B-cell loss), which leaves the patients largely defenseless against common infections. Different genes may be affected in SCID patients, including those encoding adenosine deaminase (ADA) (ADA-SCID), the interleukin-2 receptor-γ (IL2RG) (SCID-X1), and the interleukin-7 receptor α chain. Hematopoietic stem cell transplantation (HSCT) was shown to be the only curative treatment option for SCID. For patients lacking a matched donor for allogeneic HSCT, gene therapies to modify autologous hematopoietic stem and progenitor cells (HSPC) from SCID patients with corrected versions of *ADA*, *IL2RG*, or *IL7R*, respectively, are potential therapeutic options. In 1990, the very first approved gene therapy trial was accomplished in two ADA-SCID patients to whom the 1.5 kb ADA cDNA was delivered as the therapeutic cargo via an LTR-driven gammaretroviral vector into autologous T cells obtained from peripheral blood via apheresis [82]. Although only low gene transfer efficiency was achieved (around 1%), clinical efficacy was observed for both children who were able to maintain improved immune function with greatly decreased doses of polyethylene glycol (PEG)-ADA enzyme replacement therapy. Importantly, no adverse events were observed [83]. Later, transduction of CD34^+^ HSPC with an LTR-driven gammaretroviral vector using a vector backbone generated in the Baum lab to express ADA led—after failure and cessation of PEG-ADA—to stable transgene expression and polyclonal T-cell reconstitution without adverse events [84]. Other studies provided further evidence that LTR-driven gammaretroviral vectors to deliver ADA are effective in restoring normal purine metabolism without vector-related adverse events in patients for more than 13 years after treatment [85, 86]. These studies led to the market authorization of the ATMP Strimvelis in Europe. Of note, Orchard Therapeutics, the company that owns Strimvelis, recently issued a statement about a temporary hold of the clinical use of Strimvelis after one ADA-SCID patient treated with Strimvelis-modified CD34^+^ HSPC developed a lymphoid T-cell leukemia. This severe adverse event is being investigated to determine if it may be due to insertional mutagenesis caused by Strimvelis treatment as suggested by preliminary data.

As cultivation and transduction protocols for gene transfer into HSPC improved, other trials used LTR-driven gammaretroviral vectors engineered to express the wild-type *IL2RG* gene to transduce HSPC from SCID-X1 patients. While immunity was successfully restored in most patients, this gene therapy approach was unfortunately associated with significant toxicity. Thirty percent (6 of 20) of patients in two independent trials developed acute leukemias due to activation of proto-oncogene (e.g., *LMO2*, *CCND2*, *MECOM*) expression via gammaretroviral vector insertion [87–90] and reviewed here [91]. A subsequent multinational study showed the efficacy and safety of a SIN gammaretroviral vector co-developed at Hannover Medical School to deliver the *IL2RG* gene to autologous bone marrow-derived CD34^+^ cells in nine boys with SCID-X1 (NCT01410019, NCT01175239, NCT01129544) [33]. This study showed resolution of infections in seven of eight evaluable patients with similar CD3^+^ T-cell recovery kinetics as observed in earlier studies. Analyses of retroviral vector insertion sites revealed a polyclonal integration profile with less clustering of insertion sites near known proto-oncogenes (*LMO2*, *MECOM*) and no incidence of cell transformation in any of the patients to date. Thus, the data suggest an improved safety profile of the gammaretroviral SIN vector [33]. This trial served as the basis for an ongoing multicenter trial investigating safety and efficacy of lentiviral SIN vector-based therapy in SCID-X1 patients (NCT03311503, reviewed in [92]). Of therapeutic relevance, and in contrast to earlier trials in which only T-cell reconstitution was observed, recent studies using a lentiviral SIN vector with the elongation factor 1α to express a codon-optimized common γ-chain showed reconstitution of T, B, and NK lineages (NCT01306019, NCT01512888) [93, 94].

### Wiskott–Aldrich syndrome

WAS was first described by the German pediatrician Wiskott [95] and was later further characterized by the American pediatrician Aldrich et al. [96]. WAS is an X-chromosomal recessive inherited disease characterized by immunological deficiencies with reduced ability to form blood clots due to insufficient quantity and function of thrombocytes. The observation that mutations in the *WAS* (Wiskott–Aldrich syndrome protein (WASP) actin nucleation promoting factor) gene, which encodes the cytosolic protein WASP, can result in WASP variants with attenuated function and expression level made WAS patients potential candidates for gene therapy.

In the first stem-cell gene therapy for WAS patients conducted in Germany (German Clinical Trials Register number, DRKS00000330) [97, 98], ten patients were treated with autologous CD34^+^ HSPC modified with an MLV-derived LTR-driven gammaretroviral vector (CMMP backbone—a derivative of MFG pseudotyped with GALV) engineered to express WASP. Gene therapy led to reduced frequency and severity of infections and correction of thrombocytopenia. No clonal imbalances were observed initially after gene therapy. However, one of the ten treated children did not achieve stable engraftment, and seven developed acute leukemia: T-ALL occurred in six patients between 488 and 1813 days after treatment and AML evolved in one patient [98]. Thus, gene therapy was shown to correct the disease phenotype, but the therapeutic efficacy of LTR-driven gammaretroviral vectors was heavily impaired by high transformation rates in this clinical setting.

Of note, SIN lentiviral vectors designed to express WASP via an internal elongation factor short 1α (EFS1α) promoter showed similar production of the therapeutic protein (WASP) in murine and human HSPC as compared to the LTR-driven gammaretroviral vector [99]. Indeed, gene therapy of autologous HSPC with a SIN lentiviral vector using a 1.6 kb fragment of the proximal *WAS* promoter to drive expression of the WASP therapeutic transgene was shown to be feasible and safe in seven WAS patients with severe disease [34, 100]. Although one patient died of septic shock due to drug-resistant herpes viral infections, the other six patients survived with stable engraftment of functional gene-modified cells with WASP expression observed in T cells (34–84%), NK cells (14–85%), and B cells (13–55%). Importantly, no evidence of vector-related toxicities was observed, further supporting the safety of lentiviral SIN vectors for gene therapy [32, 34].

### Chronic granulomatous disease (CGD)

CGD encompasses a group of inherited diseases that affect the ability of immune cells of the myeloid lineage to generate reactive oxygen species important for destruction of ingested pathogens. CGD patients suffer from recurrent infections due to decreased immune cell function. The most predominant form of CGC is an X-linked form (X-CGD) caused by *CYBB* (cytochrome b-245 beta chain, gp91-PHOX) mutations that result in loss of phagocyte NADPH oxidase activity. There are also autosomal recessive CGD forms that result from mutation of *CYBA* (cytochrome b-245 alpha chain), *NCF1* (neutrophil cytosolic factor 1), *NCF2*, or *NCF4* (reviewed in [101]).

A gammaretroviral LTR-driven SF71-gp91-phox, based on the SF71 backbone [14, 16, 102], was redesigned to express a codon-optimized gp91-PHOX cDNA in Frankfurt. After extensive in vitro and in vivo testing, this vector was used at the Johann Wolfgang Goethe University Hospital (Frankfurt, Germany) to transduce autologous CD34^+^ cells to treat two adult X-CGD patients (NCT00564759) [24]. Bacterial and fungal infections were initially resolved in both patients, however, the number of oxidase-positive granulocytes eventually decreased even though high numbers of gene-marked cells persisted [24, 103]. Bisulfite sequencing showed increased methylation in the LTR promoter region that corresponded with transgene silencing. Retroviral insertions within the *MECOM* (*MDS1-EVI1*) locus led to clonal outgrowth of hematopoietic clones that resulted in formation of myelodysplastic syndromes (MDS) that likely evolved to AML in one patient and refractory cytopenia with multilineage dysplasia in the second patient [103].

Similar observations were made in a subsequent study in which fungal infections were resolved in two X-CGD patients who received gammaretroviral gene therapy [104]. One child developed MDS due to insertional activation of ecotropic viral integration site (*EVI1*) and signal transducer and activator of transcription 3 (*STAT3*) genes, which led to MDS. The second patient had clonal expansion of cells with a retroviral vector insertion site in the myelodysplasia syndrome 1 (*MDS1*) gene, but did not develop MDS. As gene therapy led to initial resolution of infections in both studies, the authors suggested that future studies should evaluate the therapeutic efficacy of potentially safer lentiviral SIN vectors in X-CGD patients. Thus, a lentiviral SIN vector engineered to express hGP91-PHOX from the cathepsin G/cfes promoter (a phagocyte-specific promoter) was co-developed by groups in London and Frankfurt [105]. Preclinical assessment indicated that transduced CD34^+^ cells from X-CGD patients engrafted in xenograft mouse models and showed therapeutically relevant NADPH levels in gp91-phox expressing myeloid cells with no evidence for adverse mutagenic events linked to insertional mutagenesis [106]. These studies paved the way for the approval of respective clinical trials in Europe and the USA (NCT01855685; NCT02234934). Initial results of a multinational trial evaluating efficacy of gene therapy with this lentiviral SIN vector in nine X-CGD patients were recently published [107]. Clinical efficacy was shown for more than 12 months in six out of the nine patients with no evidence of transgene silencing or clonal expansion. Preexisting morbidities led to the death of two patients within 3 months of gene therapy treatment, while one patient had only low levels of oxidase-positive neutrophils, most likely due to low engraftment levels of gene marked cells [108].

### Cerebral adrenoleukodystrophy (CALD)

CALD is an X-linked peroxisomal metabolic disorder caused by mutations in the *ABCD1* gene, which encodes the peroxisomal membrane ALD protein (ALDP). Due to loss of ALDP function, very-long-chain fatty acids accumulate in the brain, spinal cord, plasma, and adrenal glands. CALD is characterized by progressive inflammatory demyelination in the brain, which, in the absence of treatment, results in death within 10 years of the diagnosis.

The efficacy and safety of autologous CD34^+^ HSC transduced with the Lenti-D^TM^ (elivaldogene autotemcel) lentiviral vector was tested in a phase 2/3 study with 32 CALD patients (NCT01896102). The vector encodes the human adrenoleukodystrophy protein driven by a retroviral MND promoter. The trial was sponsored by bluebird bio and included a trial site at the University of Leipzig (Germany). An interim report on the trial following treatment of 17 boys with early stage CALD showed gene-marked cells in all patients with no treatment-related deaths, graft-versus-host disease (GVHD), or evidence of insertional mutagenesis after a median follow-up of 29.4 months [109]. This successful trial was presented at this year’s annual meeting of the European Society for Blood and Marrow Transplantation (EBMT abstract O077). This gene therapeutic approach was also used to treat one patient in Germany and the trial will be expanded to include more patients, including those in Germany (personal communication Dr. W. Köhler, University Clinic Leipzig). One potential advantage of using gene therapy instead of standard HSCT to treat CALD patients is that gene-modified cells express supra-physiological levels of the ALDP, which allows cross-correction of endogenous, non-modified cells. Several animal models of lysosomal storage diseases as well as clinical experience showed a clear correlation between enzyme dose and therapeutic efficacy, with increased therapeutic effects observed at higher protein levels [35, 110, 111].

### Hemoglobinopathies

Hemoglobinopathies are inherited diseases characterized by mutations and/or deletions in α- or β-globin genes, leading to defective or unstable hemoglobin synthesis. The two main groups of hemoglobinopathies are autosomal recessive thalassemia syndromes and the autosomal dominant hemoglobin disorders. Hemoglobinopathies have become more common in Germany due to steadily increasing immigration [112]. Previously, allogeneic HSCT was the only curative option for hemoglobinopathies, but today, gene therapy trials are available for patients lacking suitable HSCT donors. At least three studies sponsored by bluebird bio using lentiviral vectors to transduce autologous hematopoietic cells ex vivo are conducted at sites in Germany. For example, one ongoing study is a multicenter study aiming for long-term follow-up of hemoglobinopathy patients (β-thalassemia or severe SCD) who were treated with gene therapy (NCT02633943) with a site at the Hannover Medical School (personal communication Dr. Martin Sauer).

Another single-arm, multi-site, single-dose, phase 3 study is evaluating the efficacy and safety of gene therapy with LentiGlobin BB305 (Betibeglogene autotemcel, Zynteglo^TM^) in transfusion-dependent β-thalassemia patients ≤ 50 years old and who have a β0/β0, β0/IVS-I-110, or IVS-I-110/IVS-I-110 genotype (NCT03207009). Sites in Germany include Hannover Medical School and University of Heidelberg.

A further phase 3 single-arm multi-site study evaluates the efficacy and safety of gene therapy with a lentiviral βA-T87Q-globin vector in transfusion-dependent β-thalassemia patients who are ≤50 years old and who do not have a β0/β0 genotype (NCT02906202). Hannover Medical School participates in this trial. Encouragingly, Zynteglo recently received market authorization by European Medicines Agency (EMA). There will also be trials for sickle-cell disease with German centers.

### Junctional epidermolysis bullosa (JEB)

JEB is a skin adhesion disorder characterized by fragility of the skin and mucous membranes due to mutations in genes such as *COL17A1*, *ITGB4*, *LAMA3*, *LAMB3*, or *LAMC2* that are important for proper generation of the basement membrane. Mavilio, de Luca and colleagues [113] demonstrated the feasibility and safety of gene therapy to treat an adult patient with LAM5-β3 JEB caused by mutation of the *LAMB3* gene. Primary keratinocytes from the patient were transduced with a gammaretroviral vector engineered to express the full-length *LAMB3* cDNA via the Moloney leukemia virus LTR (MLV-LTR). LAM5 was expressed and functional in the modified skin tissue and the transplanted skin remained stable throughout the 1-year follow-up with no blisters, infections, inflammation, or immune response [113]. In the Burn Center of the BG University Hospital Bergmannsheil at Ruhr University Bochum, gene therapy was successfully used to save the life of a 7-year-old child who had a severe form of JEB, which caused blisters and skin erosion on about 80% of his total body surface area [114]. An LTR-driven gammaretroviral (MLV-RV) vector was used to express full-length *LAMB3* cDNA, and the regenerated skin produced by this gene therapy approach was resistant to mechanical stress with phenotypic correction as shown by the absence of blisters or skin erosion over 21 months. Clonal tracing showed that gene repair in holoclones, long-lived stem cells of the epidermis, was responsible for the successful, long-lasting effects. The basic stem cell concepts learned from this study are expected to help direct further innovations in gene and stem cell therapies.

## Acquired diseases including cancer and infections

### Selection and elimination approaches in gene therapy

While allogeneic HSCT is the only curative treatment for various malignant as well as nonmalignant diseases, it is commonly associated with potentially life-threatening side effects such as severe infections and GVHD. As GVHD is caused by T cell-mediated destruction of healthy tissue in the transplant recipient, use of T cell-depleted transplants can alleviate GVHD risk. However, potential benefits of antitumor (graft-versus-leukemia (GvL)) and anti-infection (graft-versus-infection (GvI)) activity of T cells are also lost with this approach. In an effort to minimize GVHD risk and maintain beneficial GVL and GVI in AML patients, allogeneic T cells were modified with a suicide gene to allow inducible depletion of gene-modified allogeneic T cells in the case of severe GVHD reactions [115, 116]. To ensure transplantation of gene-modified T cells, it is necessary to generate suitable selection markers that are not too immunogenic and that are rapidly and stably expressed. This concept was initially developed by C. Bonini et al. [115] and P. Tiberghien [116] and tested in clinical trials.

A phase 1/2 study to evaluate transplantation of CD34-enriched peripheral blood stem cells modified with the *HSV*-*TK* suicide gene expressed from the Mo3TIN (MoMLV-LTR) vector was accomplished in one MDS and two CML patients in Hamburg [117]. No acute toxicities were observed and all three patients engrafted quickly after transplantation. Stable numbers of modified T cells were observed for one patient, but were lost in the other two patients, possibly due to immune rejection in one patient. The second patient developed acute skin GVHD grade II, which was completely resolved with ganciclovir treatment and rapid loss of gene-modified T cells. Both patients who lost gene-modified T cells subsequently developed secondary graft failure, indicating the importance of donor CD3^+^ cells to promote engraftment and protect from eventual graft rejection.

To overcome the need for prolonged ex vivo selection of TK-transduced T cells with G418 as in the previous trial [117], Fehse et al. [118] identified a truncated form of CD34 (tCD34) that was used to enrich gene-modified primary human T cells expressing tCD34 to >95% purity (Fig. 1). This was then further developed to couple a splice-corrected variant of Herpes simplex virus thymidine kinase (scHSVtk), which can be used as a suicide gene as cells that express it are highly sensitive to the prodrug ganciclovir, to tCD34 to generate a tCD34–scHSVtk fusion protein [119]. High expression of the “sort-suicide” selection marker was achieved using the gammaretroviral hybrid vector MP71 containing the MPSV-LTR and MESV leader 71 sequence. This strategy was shown to be feasible and safe in three children who received T cell-depleted CD34^+^ cell-enriched mismatched allogeneic grafts (NCT01204502) [120]. MP71 vectors exhibited improved transgene expression in T cells and were also used for successful transfer of T-cell receptor (TCR) for adoptive T-cell therapies directed against cancer cells [15, 121] and were also used in several clinical trials for immunotherapies [122].

A multicenter phases 1–2 study with a clinical trial site at the Hannover Medical School investigated the infusion of donor lymphocytes transduced with the suicide gene *HSV*-*TK* in 50 high-risk leukemia patients after haploidentical stem-cell transplantation (NCT00423124) [123]. Donor lymphocytes were modified with the gammaretroviral vector (MSV/MLV-LTR) SFCMM3 that expresses HSV-tk via the LTR and contains the (SV40) low-affinity receptor for nerve growth factor from which the intracellular domain has been truncated (ΔLNGFR) to allow selection of transduced cells (Fig. 1). The safety of this gene therapy approach was demonstrated as no acute or chronic adverse events were found to be due to the gene therapy. Infusion of TK-modified lymphocytes appeared to accelerate immune reconstitution and induction of the suicide gene successfully controlled GVHD in ten patients with acute GVHD and one patient with chronic GVHD [123]. The safety and efficacy of SFCMM3-modified T cells to control GVHD were also shown by Weissinger et al. [124], who also observed stabilization of donor chimerism following infusion of the modified T cells and successful resolution of GVHD upon ganciclovir administration to eliminate modified donor T cells. In 2016, the SFCMM3-based ATMP received conditional marketing authorization (CMA) in Europe (as Zalmoxis®), but the parallel phase 3 trial (with several participating centers in Germany, including Hannover and Hamburg) was terminated and the CMA was withdrawn at the request of its holder (MolMed) for commercial reasons at the end of 2019. Preuss et al. [125] further improved the activity of this suicide gene therapy by introducing the A168H mutation into the codon-optimized scHSVtk to generate the TK.007 suicide gene. TK.007 expression mediates faster cell killing at lower ganciclovir concentrations, which should reduce nonspecific toxicity such as myelotoxicity and makes the gene particularly interesting for cancer gene therapy [126] as further discussed below.

### Gene therapy strategies to treat cancer

In addition to their use to eliminate allogeneic T cells in the case of adverse events such as GVHD, gammaretroviral- and lentiviral-mediated transfer of suicide genes can also be applied to eliminate cancer cells. In fact, this concept was one of the first gene-therapy principles tested in a large phase 3 clinical study in glioblastoma back in the 1990s [127]. Unfortunately, that study did not show clinical efficacy, most probably due to low transduction rates of tumor cells in vivo. Consequently, many efforts were directed toward improved and selective transduction of malignant brain cells. For example, lentiviral vectors pseudotyped with LCMV glycoprotein were shown to efficiently and selectively transduce solid glioma and infiltrating tumor cells [44]. Use of lentiviral vectors pseudotyped with LCMV-G or VSV-G to deliver HSV-tk led to complete remission of solid tumors in a glioblastoma xenograft model [128].

### Modified T cells to treat cancer

As discussed above, gene-modified T cells can be used in transplantation settings to protect patients against unwanted effects like GVHD, but allogeneic T cells can also elicit graft-versus-tumor activity. Building upon these principles, T cells were engineered to express TCRs or chimeric antigen receptors (CARs) designed to specifically target tumor-associated antigens, or when possible, neoantigens. These technologies exploit inherent T-cell activities and redirect the cytotoxic potential of T cells to improve tumor cell recognition and elimination. Clinical efficacy of TCR- and CAR-modified T cells is currently being evaluated in hundreds of studies worldwide, including trials in which German investigators participate as collaboration partners and/or principal investigators (Table 1). German researchers, e.g. Abken, Rössig, Blankenstein, and Uckert, have made significant contributions to basic and translational CAR and TCR research [121, 129–142], including lentiviral SIN EFS1α and LTR-driven gammaretroviral vector systems. Recently, different German groups developed methods for high-sensitivity in vivo monitoring of CAR T cells [143–145].

**Table 1:** Selected CAR- and TCR-modified T-cell trials with clinical testing sites in Germany.

Disease	Sponsor	Trial sites in Germany	Trial number
CAR T-cell studies			
Hematologic and lymphatic malignancies positive for CD123	Cellex Patient Treatment GmbH	University Hospital Ulm University Hospital Würzburg Philipps-University Marburg University Hospital Dresden University Hospital Leipzig	NCT04230265
Relapsed/Resistant CD20 positive B-NHL	Miltenyi Biomedicine GmbH	University Hospital Cologne University Hospital Leipzig	NCT03664635
MM	Janssen Research & Development, LLC	University Hospital Hamburg Eppendorf University Hospital Heidelberg University Hospital Würzburg	NCT04133636
Metastatic melanoma	Miltenyi Biomedicine GmbH and DLR German Aerospace Center	University Hospital of Cologne	NCT03893019
Relapsed/refractory CD19 positive B-cell malignancies	Miltenyi Biomedicine GmbH	University Hospital Erlangen University Hospital Münster	NCT03853616
Relapsed and lenalidomide-refractory MM	Janssen Research & Development, LLC	University Hospital Carl Gustav Carus Dresden at the Technical University of Dresden University Hospital Hamburg Eppendorf University Hospital Heidelberg University Hospital Cologne University Hospital Leipzig Eberhard-Karls-University Hospital University Hospital Würzburg	NCT04181827
Relapsed/refractory MM	Celgene	University Hospital Heidelberg University of Tübingen University Hospital Würzburg	NCT03361748
Relapsed/refractory MM (KarMMa-3)	Celgene	University Hospital Heidelberg University Hospital Cologne University Hospital Würzburg	NCT03651128
Relapsed/refractory DLBCL	Novartis Pharmaceuticals	Novartis investigative sites (Cologne, Würzburg)	NCT02445248
Refractory/relapsed FL	Novartis Pharmaceuticals	Novartis investigative sites (Cologne, Münich, Ulm)	NCT03568461
Relapsed/refractory CLL	Kite, A Gilead Company	University Hospital Heidelberg	NCT03624036
Relapsed/refractory DLBCL or other aggressive B-cell malignancies	Celgene	Medizinische Klinik und Poliklinik I (Dresden) University Hospital Heidelberg University of Cologne MU Klinikum der Universität (Munich) Universitatsklinikum Ulm	NCT03484702
Refractory aggressive NHL	Kite, A Gilead Company	University Hospital Dresden University Hospital of Essen University Hospital Würzburg	NCT02348216
Relapsed/refractory DLBCL (ZUMA-7)	Kite, A Gilead Company	University Hospital Dresden University Hospital Gottingen University Hospital Hamburg-Eppendorf University Hospital Heidelberg University Hospital Munster University Hospital Würzburg	NCT03391466
MB-CART2019.1 in patients with relapsed or resistant CD20 and CD19 positive B-NHL	Miltenyi Biomedicine GmbH	University Hospital Augsburg University Hospital Cologne University Hospital Hamburg-Eppendorf	NCT03870945
Tisagenlecleucel versus standard of care in adult patients with relapsed or refractory aggressive B-NHL (BELINDA)	Novartis Pharmaceuticals	Novartis investigative sites (Regensburg, Berlin, Hamburg, Cologne, Leipzig, Münich, Ulm)	NCT03570892
JCAR017 compared to standard of care in adult patients with high-risk, second-line, transplant-eligible relapsed or refractory aggressive B-NHL (TRANSFORM)	Celgene	Robert-Rössle-Clinic in HELIOS Clinic Berlin-Buch Clinic University Hospital Carl Gustav Carus Dresden University Hospital Hamburg-Eppendorf University of Cologne University Hospital Munster LMU Clinic at University of Münich	NCT03575351
Safety and efficacy of allogeneic CRISPR-Cas9-engineered T cells (CTX110) in patients with relapsed or refractory B-cell malignancies (CRSP-ONC-001)	CRISPR Therapeutics AG	University of Hamburg	NCT04035434
TCR-modified T-cell studies			
High-risk myeloid and lymphoid neoplasms (CD-TCR-001)	Medigene AG	University Hospital Dresden University Hospital Erlangen University Hospital Frankfurt University Hospital Freiburg University Hospital Heidelberg University Hospital Leipzig University Hospital Mainz University Hospital Regensburg University Hospital Würzburg	NCT03503968
Adult solid tumors	Immatics US, Inc.	University Hospital Würzburg University Hospital Bonn University Hospital C.-G.-Carus Dresden	NCT03441100
WT1 TCR therapy in MDS or AML patients who failed to achieve or maintain an IWG response following hypomethylating agent therapy	Cell Medica Ltd	University Hospital Dresden	NCT02550535

*AML* acute myeloid leukemia, *B-NHL* B-cell non-Hodgkin lymphoma, *CLL* chronic lymphocytic leukemia, *DLBCL* diffuse large B-cell lymphoma, *FL* follicular lymphoma, *IWG* international working group, *MDS* myelodysplastic syndrome, *MM* multiple myeloma, *NHL* non-Hodgkin lymphoma.

Thus far, at least three market-authorized CAR T-cell drugs are available, two that use an MSCV LTR-driven gammaretroviral vector for CD19-CAR (CD28-CD3ζ) transfer (Yescarta® = Axicabtagene ciloleucel, Tecartus® = Brexucabtagene autoleucel/currently only Food and Drug Administration (FDA) approved) and one that uses a SIN lentiviral vector to modify T cells with the CD19-CAR (CD8α-4-1BB-CD3ζ) expressed from an internal EF-1α promoter (Kymriah® = Tisagenlecleucel). Interestingly, and in support of observations from the early ADA-SCID trial [82], no transformation events due to retroviral gene transfer have been observed even when LTR-driven gammaretroviral vectors were used to express CAR constructs. This may be due to the resilience of mature somatic cells such as T cells to transformation as compared to stem cells [21, 22].

While CAR T cells were shown to be effective in liquid tumors derived from lymphoid hematopoietic lineages, solid tumors have presented additional challenges to CAR T cells therapies. One strategy to improve the antitumor activity of CAR T cells was to incorporate a second gene expression cassette that was controlled by nuclear factor of activated T cells signaling, which was activated upon recognition of target antigen by the CAR. Chmielewski et al. [130] and Chmielewski and Abken [138] named these “T cells redirected for unrestricted cytokine-initiated killing” (TRUCKs) and showed that IL-12 and IL-18 were delivered to tumor sites with improved antitumor response due to recruitment of additional immune cells. The TRUCK approach originally used a two vector system, one to deliver the CAR and one to deliver the inducible expression cassette, but was recently converted to a single vector system that should ease further clinical development of this gene therapy concept [146]. The CAR principles have also been successfully transferred to other immune cells such as NK cells, and CAR-NK cells were shown to have potent anticancer activity [147–151].

### Gene therapy to protect cells from HIV-1 infection

Discovery of the mechanisms of viral entry into target cells can also be exploited to protect cells from viral infection, such as HIV-1. For example, the gp41 subunit of the HIV-1 envelope glycoprotein gp120 was used as a target to inhibit HIV-1 infection. While gp120 binds target cell receptors and thus determines viral tropism, gp41 mediates fusion of the viral and target cell membranes [152]. LTR-driven expression of a membrane-anchored version of T20 (also known as DP178, C36), a 36-amino acid C peptide corresponding to amino acids 638–673 of HIV_HXB2_gp41 that potently inhibits HIV-1 cell entry by locking gp41 in a conformation that does not allow fusion of the viral lipid membrane with the target cell plasma membrane, via gammaretroviral vectors was shown to protect cell lines from HIV-1 infection [152, 153]. This strategy developed by the von Laer group was further improved to minimize immunogenic side effects and membrane-anchored C peptides T20 (C36) and C46 (corresponding to amino acids 628–673 of gp41) were shown to effectively inhibit HIV-1 infection of human primary blood lymphocytes and C46 efficiently blocked entry of C36-resistant HIV-1 variants [154]. In another advance, the HIV-1 entry inhibitor derived from gp41 was cloned into a lentiviral SIN vector using a CMV promoter to express membrane-bound C46 [155]. Delivery of the entry inhibitor using this “safer” vector system to transduce primary human T cells protected modified T cells from infection with the CXCR4-tropic HIV strain BK132 [155].

A clinical phase 1 study used the LTR-driven gammaretroviral vector M87o [154] to express membrane-anchored C46 in autologous T cells, which were infused into ten HIV-infected patients who had advanced disease and were failing highly active antiretroviral therapy (HAART) [156]. T-cell transduction efficiencies ranged from 6.3 to 21.7% and no major toxicities were observed. However, a clinical improvement due to the gene-modified T cells was also not clearly demonstrated as the study protocol allowed changes in the HAART treatment 3 months after T-cell infusion [156]. A follow-up trial using the M87o vector to modify autologous CD34^+^ peripheral blood progenitor cells mobilized from HIV-1^+^ acquired immune deficiency syndrome (AIDS) patients with cancer had to be stopped after treatment of three patients due to the adverse events observed in the SCID and CGD trials described above (NCT00858793).

A preclinical study in a nonhuman primate model of HIV-1 and a chimeric simian immunodeficiency virus/HIV-1 infection demonstrated the feasibility of using an SFFV promoter-driven lentiviral vector to modify HSC for transplantation into AIDS patients scheduled to undergo chemotherapy [157]. Here, the authors incorporated a mutant methylguanine methyltransferase (MGMT^P140K^) to endow resistance of modified HSC and their progeny to chemotherapeutic challenge, which resulted in an in vivo enrichment of gene-modified HIV-resistant cells expressing entry inhibitor C46 [157] (Fig. 1).

Development of novel strategies continues to be an important effort in the gene therapy field, especially due to the possibility to avoid side effects of current standard HIV treatments as well as to offer treatment options for those patients whose infections do not respond to HAART. Furthermore, gene therapy could offer a one-time treatment with potential cure, which makes this strategy attractive to patients as well as the health care system. The occurrence of naturally resistant CCR5-negative cells, which were successfully transplanted in allogeneic settings and led to complete long-term elimination of HIV in AIDS patients [158], strongly supports evaluation of the potential for broader application of gene therapy in this disease (Fig. 1). Furthermore, the possibility to use gene therapy to excise the HIV-1 proviral sequence from the genome of infected cells was shown as discussed in more detail below [159, 160].

## Next-generation gene therapy tools

Recent advances in gene editing have created a new generation of tools for gene therapy, i.e., molecular scissors to engineer the genome in a targeted and tailored fashion. To achieve this, different gene-editing tools were created, which are based on zinc-finger nucleases [161], transcription activator-like effector nucleases (TALENs) [162, 163], and clustered regularly interspaced short palindromic repeats (CRISPR)-Cas9-based nucleases [164]. While the former two exploit DNA-binding protein domains, which are linked to a *Fok*I endonuclease, the CRISPR-Cas9 system has given the genome-editing field a new boost and takes advantage of an RNA-guided nuclease mechanism. All three concepts are currently used in clinical applications worldwide, either to generate a KO by DNA scission and nonhomologous end-joining repair or alternatively by DNA scission and homologous recombination, if a suitable DNA donor for repair is available.

The first patient receiving CRISPR-Cas9-modified HSC for globinopathies was treated in Germany, in a CRISPR Therapeutics and Vertex Pharmaceuticals sponsored investigational clinical trial based on autologous, gene editing-mediated HSCT for the treatment of severe transfusion-dependent beta thalassemia (TDT) and SCD (CTX-001) (NCT03745287, NCT03655678). The respective designer nuclease was designed to target and suppress the erythroid-specific enhancer of the *BCL11A* gene, which results in an upregulation of HbF, the fetal hemoglobin. Increased HbF levels are associated with decreased severity in TDT and SCD, as learned from the clinically used HbF inducer hydroxyurea. First promising results were presented by Selim Corbacioglu (Regensburg University Hospital) at the American Society of Hematology (ASH) and the European Hematology Association (EHA) meetings, demonstrating clinically relevant increases in HbF and total Hb with a follow-up of more than 15 months without observations of major side effects related to the gene therapy intervention and no need for transfusions after treatment. Remarkably, CTX-001 has received FDA Regenerative Medicine Advanced Therapy (RMAT) designation for the treatment of severe hemoglobinopathies as well as Orphan Drug Designation from the FDA for TDT and from the EMA for SCD and TDT.

In addition to classical designer nucleases, also artificially designed and evolved recombinases may contribute to the arsenal of next-generation tools for gene editing. Hauber (Hamburg) and Buchholz (Dresden) have taken a very interesting route in this regard. Starting with the loxP-recognizing Cre recombinase, they generated the Tre recombinase through molecular evolution. Interestingly, Tre recognized a defined target sequence in the LTR of the integrated HIV-1 provirus, resulting in the excision and eradication of HIV-1 from infected cells. In further work, they demonstrated accurate provirus excision, noteworthy in the absence of cytopathic effects, in Tre-transduced T helper cells and HSPCs after transplantation into humanized mice [159]. As this would only cover a small proportion of HIV-1 strains, the authors further evolved a broad range recombinase (Brec1) that recognizes the majority of clinically relevant HIV-1 strains and subtypes [160]. Encouragingly, Brec1 efficiently removed integrated proviruses from patient cells infected with clinical HIV-1 strains in vitro and in vivo in humanized mouse models. A first-in-human phase 1b/2a gene therapy trial targeting HIV-1 by Brec1-mediated genome editing (HIVCURE [165]) is currently under development at the University Medical Center Hamburg-Eppendorf to evaluate safety and HIV-1 provirus excision efficacy. HIV-infected lymphoma patients undergoing chemotherapy (with rituximab, cyclophosphamide, doxorubicin, vincristine, and prednisolone) will be additionally treated by autologous transfer of Brec1-transduced CD34^+^ HSPCs.

In addition to tailored gene editing, also other next-generation principles may be considered, which include more randomly integrating safer vectors as well as non-integrating retroviral vectors. Interestingly, in the rich world of retroviruses, different virus family members have evolved, which have a more random integration pattern, e.g., RSV-derived alpharetroviruses, human T-lymphotropic virus 1 and, to a lesser degree, Foamy viruses [166]. Based on the wild-type avian sarcoma-leukosis virus (ASLV), Hughes [167] constructed a replication-competent ASLV-derived retroviral vector, which was an excellent tool to better understand retrovirus biology of ASLV and retroviruses in general. In addition, Suerth et al. [79, 80] generated an ASLV SIN vector system using a split-packaging system (i.e., vector, gag/pol, and envelope sequences were on separate plasmids to avoid recombination) and production in human 293-derived vector producer cells. Interestingly, this vector system preserved the close-to-random integration preference [79, 81], had only a low genotoxicity, and efficiently transduced HSPCs, T cells, and NK cells [150, 168–170]. Furthermore, Kaufmann et al. [171] demonstrated that alpharetroviral SIN vectors were capable of correcting human X-CGD CD34^+^ HSPCs upon transplantation into a humanized mouse model. In contrast to lentiviral vectors, no aberrant splicing was found, which underlines the lower genotoxicity.

Foamy viral vectors are another class of retrovirus vectors, which were deeply explored and developed in Germany, mainly by Rethwilm and Lindemann. Foamy viruses are interesting in that they also have a relatively random integration pattern, seem to be apathogenic in their hosts, and their replication strategy uses a combination of features employed by retroviruses and hepadnaviruses [172]. A split-packaging system was generated, with reduced *cis*-acting sequences [173]. Noteworthy, foamy viral vectors integrated less frequently near RefSeq genes and proto-oncogene transcriptional start sites in a humanized CD34^+^ cell transplantation model [174] and, encouragingly, were efficiently used to correct leukocyte adhesion deficiency in dogs [175]. Moreover, direct in vivo delivery of foamy vectors enabled correction of canine SCID-X1 [176].

In addition to integrating retroviral vectors with an intrinsically safer integration profile, also non-integrating retroviral vectors deserve further attention. By directed modulation of defined retroviral steps of the life cycle, these non-integrating tools can be engineered to express (a) circular 1- and 2-LTR episomes, (b) retroviral or non-viral mRNAs as well as (c) defined proteins. These represent interesting tools for settings in which a designer nuclease or recombinase can be transiently expressed to mediate genome modification in target cells.

## Conclusions/outlook

The gene therapy field has certainly faced many challenges in the past, but systematic appraisal of expected as well as unexpected outcomes led to improved understanding of mechanisms that govern cell transformation and how to minimize the risk of adverse events in gene therapy patients. Thus, clinical experience has directly translated into successful development of safer vectors and treatment protocols. As we continue to learn the intricacies of retroviral-mediated gene transfer, including cell targeting, entry mechanisms, control of genome insertion sites, and transgene expression, gene therapy protocols will certainly become even safer and more efficacious. While there has been a great amount of progress in the field of gene and cell therapy, it is of utmost importance that we continue to build upon this enthusiasm to further develop gene therapy capacities in order to be able to deliver these life-saving therapies to as many of the patients in need as possible. To accomplish this, efforts should be directed toward advanced production capacities, identification of additional patients who could benefit from these treatments, improved safety, follow-up, and monitoring. These goals will require the continued international cooperations that have positioned the field so well to date. Thus, we look forward to further development of the national and international networks as well as supporting infrastructures needed to continue to promote gene therapy.
